# Species Diversity in the Leafhopper Genus *Batracomorphus* Lewis (Hemiptera: Cicadellidae: Iassinae) from Qinling Mountain in Shaanxi

**DOI:** 10.3390/insects12060494

**Published:** 2021-05-26

**Authors:** Yulin Hu, Lili Gao, Zihan Han, Wu Dai

**Affiliations:** Key Laboratory of Plant Protection Resources and Pest Integrated Management of the Ministry of Education, College of Plant Protection, Northwest A&F University, Xianyang 712100, China; hyl18629500557@gmail.com (Y.H.); 2018050227@nwafu.edu.cn (L.G.); hzh17392448255@nwafu.edu.cn (Z.H.)

**Keywords:** Auchenorrhyncha, Iassinae, Batracomorphini, morphology, distribution, Qinling

## Abstract

**Simple Summary:**

Qinling Mountain is one of the global biodiversity hotspots, dividing northern temperate zones from subtropical zones. However, no species of *Batracomorphus* was previously recorded from Qinling Mountain in Shaanxi. We collected some specimens of *Batracomorphus* from this site and classified these into nine species based on a comparative morphological study, including one new species and one new synonym. The results of this study revealed species diversity and distributions of *Batracomorphus*.

**Abstract:**

The genus *Batracomorphus* Lewis is the third largest leafhopper genus in the world, with its greatest diversity in the Oriental region. Here, nine species of *Batracomorphus,* including one new species, are recorded from Shaanxi Province, China, for the first time: *B. allionii* (Turton), *B. clavatus* Cai and Shen, *B. fletcheri* Hu and Dai sp. nov., *B. geminatus* (Li and Wang), *B. juno* Knight, *B. lateprocessus* Li and Wang, *B. lunatus* Cai and He, *B. subfuscus* (Li and Wang) and *B. pandarus* Knight. Among them, *B. juno* Knight is recorded from China for the first time. One new synonym is revealed: *B.* *nigromarginattus* Cai and Shen, 1999 is a junior synonym of *B. subfuscus* (Li and Wang, 1993). All taxa are described, and photographs of male genitalia are given based on observations of specimens from Qinling Mountain in Shaanxi of China. A key to the species found in Qinling Mountain of Shaanxi is also provided.

## 1. Introduction

The Cicadellidae is the largest family in the Hemiptera with more than 23,000 described species distributed worldwide. Iassinae is one of 25 subfamilies of leafhoppers and comprises more than 2000 species and 154 genera currently placed in twelve tribes [1,2,3,4,5]. Iassines are mostly arboreal and are distributed worldwide, but most of the tribes and genera are restricted to a single biogeographic realm.

The widespread Old-World genus *Batracomorphus*, originally established by Lewis (1834) [6], is the third largest leafhopper genus in the world and contains ca. 360 species, distributed throughout the temperate and tropical regions of the Eastern Hemisphere. Species of *Batracomorphus* are mainly arboreal, although some also occur on shrubs, herbaceous plants, and grasses [7,8]. Some species of this group cause injury to economically important trees, such as willow, apples, pears, and other fruit trees, as well as to alfalfa, rice and other crops [9]. In China, *Batracomorphus allionii* damages economic trees [10] and feeds on bamboo [11], and *B. pandarus* injures wine grapes [11]. Elsewhere, *Batracomorphus angustatus* (Osborn) is a vector of the phytoplasmas which cause tomato big bud and potato purple top wilt disease [12]. *Batracomorphus angustatus* is a possible vector of Australian grapevine yellows [13].

Reviews of the species of *Batracomorphus* from the Afrotropical region [14,15], Oriental region [8,16], and Australian region [8] have revealed the high diversity of the genus and provide distributional data for regional faunas, but more comprehensive studies are still needed to elucidate the degree of overlap among regional faunas.

China is one of the world’s most biodiverse countries, hosting more than ten percent of known species [17], including a considerable number of endemic species. Until now, 36 species of *Batracomorphus* have been described or recorded in China, many of which are apparently endemics [18,19,20,21]. Shaanxi is a north-central province of China, and includes portions of the Loess Plateau in the middle reaches of the Yellow River, as well as the Qinling Mountain across the southern part of the province. The latter is one of the global biodiversity hotspots, dividing northern temperate zones from subtropical zones. Due to the diverse climate and geographic conditions, hundreds of new insect taxa, including some new genera, have been described from Shaanxi in recent years [22,23,24,25,26]. However, the diversity of *Batracomorphus* has never been studied thoroughly and no species of *Batracomorphus* was previously recorded from Qinling Mountain in Shaanxi.

During our ongoing review of Chinese Iassinae, we discovered many specimens of *Batracomorphus* from Qinling Mountain in Shaanxi. In this paper, we review the known species of *Batracomorphus* from Shaanxi and describe one new species, *B. fletcheri* sp. nov. Moreover, the previously known species (*B. allionii* (Turton), *B. clavatus* Cai and Shen, *B. geminatus* (Li and Wang), *B.* *juno* Knight, *B. lateprocessus* Li and Wang, *B. lunatus* Cai and He, *B. subfuscus* (Li and Wang) and *B.* *pandarus* Knight) are redescribed because their original descriptions were very brief. A checklist of the nine species from Shaanxi is provided together with a key for their separation.

## 2. Materials and Methods

Specimens examined were collected using sweep nets and light traps. In the present study, 69 specimens of the genus *Batracomorphus* were collected from Qinling Mountain in Shaanxi and other materials were collected from several provinces in China, as shown in the following section. All specimens, including type specimens, were deposited in the Entomological Museum of Northwest A&F University (NWAFU), Yangling, Shaanxi, China.

The identification of specimens was based mainly on morphological comparisons of male genitalia. Abdomens were removed from all male specimens and boiled for about ten minutes or soaked in 5~10% NaOH for 24 h, to dissolve the muscle. They were then washed in distilled water and transferred to glycerin for further dissection and observation. Female specimen identification was based on comparisons of the morphological features of conspecific male specimens that were obtained at or near the same collection site. Specimen micrographs were taken using OLYMPUS PM-10AD (Olympus Co. LTD, Tokyo, Japan) and Nikon AFX-II stereomicroscopes (Nikon Imaging Japan Inc., Tokyo, Japan) with a Q Imaging digital camera (QImaging, Surrey, BC, Canada), captured in Q-Capture Pro 7 (QImaging, Surrey, BC, Canada) and compiled in Auto-Montage Pro (Synoptics Ltd., Cambridge, UK). After observation, the dissected genitalia were stored in a micro vial containing fresh glycerol. The resulting images were compiled and prepared as plates, using Adobe Photoshop CS2 (Adobe Systems, San Jose, CA, USA).

## 3. Results and Discussion

### 3.1. Generic Character

*Batracomorphus* Lewis

*Batracomorphus* Lewis, 1834: 51 [6]. Type species: *Batracomorphus irroratus* Lewis, 1834: 52 [6].

*Acojassus* Evans, 1972: 656 [16]. Type species: *Acojassus montanus* Evans, 1972 [16].

Medium-sized (4–8 mm) leafhoppers with overall usually pale green (fading to yellow in dry specimens), rarely with brown markings. Head slightly narrower (rarely equal to or wider) than pronotum. Vertex in dorsal aspect short, transversely striate; length uniform, rarely slightly longer medially; anterior margin broadly rounded in dorsal aspect; in lateral aspect slightly declivous in line with pronotum, continuously curved to face or with dorsal area flattened. Face short, broad; maxillary plates wide, lateral margins sinuate. Lora widely separated from margin. Frontoclypeus broad, approximately circular in outline, laterofrontal sutures terminating just above antennal ledges. Clypellus distinct, short, broad, sides parallel. Antennae near ventral margin of eyes. Antennal ledges prominent, transverse or slightly oblique, extending onto frontoclypeus. Antennal pits deep. Ocelli distinct, near anterior margin of face, each approximately midway between midline and corresponding eye. Pronotum longer than vertex, slightly declivous anteriorly, increasing in width posteriorly, rarely parallel-sided; lateral margins long, strongly carinate; posterior margin shallowly concave; transversely striate. Scutellum long. Forewings long, exceeding abdomen, semi-coriaceous with appendix and first apical cell membranous, weakly punctate, rarely with small tubercles; appendix wide; vein separating appendix and first apical cell complete throughout its length; three subapical cells, closed basally. Setal formula of hind femur III: 2:1:1.

Male genitalia with pygophore longer than height, emarginate dorsally to near mi-length; lateral lobes broadly or narrowly rounded posteriorly, short spine-like setae scattered over posterior half; elongate, arched, posteriorly directed process arising near base of ventral margin, rarely absent. Tenth segment membranous. Valve small, fused to pygophore. Sub-genital plates slender, triangular, extending to apex of pygophore, with short basal stem, the latter sometimes small or obscure; long hair-like setae usually arranged as a dorsolateral group at base, a short uniseriate row on dorsolateral margin and a multiseriate row on ventral margin extending also over medial surface of plate, the dorsolateral or ventral rows sometimes absent, plates sometimes densely setose. Eighth sternite longer than preceding ones and covering basal half of genital capsule. Style with apical process elongate, terminating in a small dorsally directed hook, a ventral subapical expansion sometimes present; lateral lobe well-developed; basal arm very short. Connective Y-shaped with anterior arms linked to each other dorsally and ventrally by plate-like extensions; articulated to aedeagus. Aedeagus simple, shaft directed dorsally, with or without process; gonopore usually long and extending over distal half of posterior margin of shaft, sometimes short; a longitudinal incision on anterior margin of shaft approximately same length as gonopore usually present.

Distribution. *Batracomorphus* occurs in all geographical regions, with the exception of the New World.

Notes. The genus *Batracomorphus*, established with *Batracomorphus irroratus* Lewis as the type species, is distributed throughout the temperate and tropical regions of the Eastern Hemisphere. Recently, *Batracomorphus* was placed in the monobasic tribe Batracomorphini. This tribe was placed as sister to Hyalojassini with strong branch support based on phylogenetic analysis, combining morphological and molecular data from multiple gene regions [5]. In that study, the newly established tribe was defined mainly on external characters: small to medium-sized, pale green or stramineous leafhoppers; head rounded in profile, without distinctly delimited crown; forewing appendix well-developed but not extended around wing apex; hindwing veins R4 + 5 and M1 + 2 completely confluent distally; male pygofer with stout setae scattered over distal half, ventral process slender, elongate, apex not falcate; sub-genital plate not completely concealed by pregenital sternite, extended nearly as far posterad as pygofer apex; female first valvulae with dorsal sculpturing strigate; second valvulae with two to three widely spaced dorsal teeth.

In external morphology, most species of *Batracomorphus* are remarkably uniform, differing only slightly in overall size and proportions. Thus, they are difficult to identify without studying the male genitalia. A comparative study of the male genitalia of available specimens revealed considerable variation in several structures, including pygophore processes, style and aedeagus, and such variation has supported separation of most of the known species. These structures were used previously to elucidate the natural relationships of the species in the Oriental and Australian regions and were divided into five informal groups [8]. All features listed above may need to be taken into consideration for accurate classification, and other evidence may be needed to further confirm species limits.

*Batracomorphus* Lewis appears to be of relatively recent origin with a broad ancestral range, arising between middle Eocene and late Miocene [5,14]. Now, it is one of largest genera in Cicadellidae with over 360 species, and predominantly occurs in the Oriental region [8]. Its major diversification has taken place in the tropics, where 121 species have been described from the Afrotropical region [14]. Recently, a number of additional species have subsequently been added to this genus. More general investigations and more taxonomic sampling are needed to reveal the biodiversity from constituent areas in the Oriental region.

### 3.2. Overview of Species Identification, Distribution

Nine species of the leafhopper genus *Batracomorphus* from Qinling, Shaanxi are recorded based on comparative morphological study, including one new species and one new synonym as shown in the following section. (Figure 1, Figure 2, Figure 3, Figure 4, Figure 5, Figure 6, Figure 7, Figure 8, Figure 9, Figure 10, Figure 11, Figure 12 and Figure 13).

#### 3.2.1. Checklist of the Species of Batracomorphus from China

  *Batracomorphus allionii* (Turton) China (Heilongjiang, Jilin, Liaoning, Inner Mongolia, Shaanxi, Zhejiang, Henan, Hubei, Anhui, Shandong, Yunnan, Guizhou, Sichuan), Austria, Azerbaijan, Belgium, Czech, Slovakia, Denmark, Finland, France, Germany, Italy, Kazakhstan, Latvia, Lithuania, Mongolia, Netherlands, Norway, Poland, Romania, Russia, Spain, United Kingdom, Ukraine.
  *Batracomorphus chlorophana* (Melichar) China (Guizhou, Taiwan, Hubei), Africa, Borneo, Burma, India, Java, Marquesas Islands, Philippines, Samoa, Sri Lanka, West Malaysia.
  *Batracomorphus clavatus* Shen and Cai China (Shaanxi, Henan).
  Batracomorphus cornutus Li China (Hainan).
  Batracomorphus dentestyleus Li China (Yunnan).
  *Batracomorphus expansus* (Li and Wang) China (Guizhou).
  *Batracomorphus extentus* Cai and He China (Zhejiang).
  *Batracomorphus fletcheri* Hu and Dai sp. nov. China (Shaanxi, Zhejiang).
  *Batracomorphus furcatus* Li China (Yunnan, Guizhou).
  *Batracomorphus fuscomaculatus* (Kuoh) China (Yunnan, Guizhou).
  *Batracomorphus geminatus* (Li and Wang) China (Shaanxi, Guizhou, Hainan).
  Batracomorphus gracilidensus Li China (Hainan).
  Batracomorphus gracilis Li China (Xinjiang).
  Batracomorphus guzhangensis Li China (Hunan).
  *Batracomorphus imitans* Jacobi China (Fujian).
  *Batracomorphus inachus* Knight China (Henan), Philippines.
  *Batracomorphus indicus* (Lethierry) China (Hubei, Sichuan, Yunnan, Taiwan), Philippines, Sri Lanka, India, Myanmar, Burma, Flores, Krakatau, Lombok, Seychelles, Sumbawa.
  *Batracomorphus irroratus* Lewis China, Afghanistan, Albania, Armenia, Austria, Azerbaijan, Belgium, Bohemia, Bulgaria, Czechia, Slovak, Denmark, France, Georgia, Germany, Greece, Hungary, Italy, Kazakhstan, Kyrgyzstan, Lithuania, Moldova, Mongolia, Moravia, Poland, Russia, Serbia, Slovakia, Switzerland, Tajikistan, Turkey, Turkmenia, Ukraine, United Kingdom, Uzbekistan.
  *Batracomorphus juno* Knight, 1983 rec. nov. China (Shaanxi, Guizhou), Philippines, Borneo, and West Malaysia.
  *Batracomorphus laminocus* Cai and He China (Zhejiang).
  *Batracomorphus lateprocessus* Li China (Shaanxi, Zhejiang, Hunan).
  *Batracomorphus lineatus* Shen and Cai China (Henan).
  *Batracomorphus lunatus* Cai and He China (Guizhou, Shaanxi, Zhejiang).
  *Batracomorphus matsumurai* (Metcalf) China (Zhejiang, Hennan, Guizhou, Sichuan, Taiwan), Japan, North Korea.
  *Batracomorphus notatus* (Kuoh) China (Yunnan, Guizhou).
  *Batracomorphus pandarus* Knight China (Xinjiang, Shaanxi,), Borneo.
  *Batracomorphus paradentatus* (Li and Wang) China (Yunnan, Guizhou).
  Batracomorphus pianmaensis Li China (Yunnan).
  *Batracomorphus punctatus* Li and Wang China (Guizhou).
  *Batracomorphus reflexus* (Kuoh) China (Yunnan).
  *Batracomorphus rinkihonis* (Matsumura) China (Zhejiang, Henan, Taiwan).
  *Batracomorphus spadix* Shen and Cai China (Henan).
  *Batracomorphus strictus* Li China (Hainan, Guizhou).
  *Batracomorphus subfuscus* (Li and Wang) China (Shaanxi, Henan, Guizhou, Zhejiang).
  *Batracomorphus trifurcatus* Li China (Hainan, Zhejiang, Guizhou).
  *Batracomorphus viridulus* (Melichar) China (Henan, Zhejiang, Sichuan, Yunnan, Huazhong), Japan, Korea.
  *Batracomorphus xinxianensis* Cai and Shen China (Henan).

#### 3.2.2. Key to Species of Batracomorphus Based on Males from Qinling Mountain in Shaanxi, China

1. Forewing without fine hair and punctation (Figure 2 and Figure 4A,B)…………………….2
  - Forewing with obvious fine setae or punctation (Figure 3 and Figure 4D,E)…………………………………………………………………………………………6
2. Aedeagus base strongly enlarged and expanded ventrally (Figure 10D), pygophore process nearly straight, thin (Figure 10A,B)………………*B. lateprocessus* Li and Wang
  - Aedeagus base not expanded ventrally, pygophore process not straight……………3
3. Style internal rim serrated subapically (Figure 5D); pygophore process slightly bifid at apex (Figure 5C), aedeagus with rounded lateral flange at apex (Figure 5E) …………………………………………………………………………*B. allionii* (Turton)
  - Style internal rim not serrated subapically………………………………………………4
4. Pygophore process with ventral rim serrate subapically, approximately as long as pygofer (Figure 7A,B); aedeagus concave in centre and oval in ventral view (Figure 7D).……………………….………………………………..……*B. subfuscus* (Li and Wang)
  - Pygophore process with ventral rim not serrated subapically…………………………5
5. Style stout with subapical ridge, apex hooked laterad (Figure 6C); aedeagus shaft with lateral margins distinctly concave in ventral view (Figure 6F)….…*B. pandarus* Knight
  - Style slender without subapical ridge, apex hooked mesad (Figure 11C); aedeagus shaft with sides triangularly produced laterad in ventral view (Figure 10E)…………………………………………………………………*B. clavatus* Cai and Shen
6. Style with hooked apex, slightly expanded proximally (Figure 9C); pygophore process with small spine on ventral rim subapically, heel-like (Figure 9B); aedeagus shaft slender, apex transparent (Figure 9D,E)………………………………*B. fletcheri* sp. nov.
  - Style apex not hooked ………………………………………………………………………7
7. Style apex bifid with finger-like lateral and triangular mesal projection (Figure 12C); pygophore process apex bifid with branches widely divergent (Figure 12B); aedeagus apex with triangular lateral extensions in ventral view (Figure 12D) …….………….…………………………………………………*B. lunatus* Cai and He
  - Style not bifid at apex, other features not as above………………………………………8
8. Style broadened preapically then abruptly narrowed with apex hooked dorsally (Figure 8C); pygophore process sinuate, tapering to acute apex, with small teeth subapically (Figure 8B,E); aedeagus shaft evenly curved and tapered from base to apex in lateral view (Figure 8F)……………………………………………*B. juno* Knight rec. nov.
  - Style slightly and evenly tapered with T-like apex (Figure 13C,E); aedeagus shaft bent posterad preapically in lateral view, apex bifid with processes extended laterad (Figure 13D,F)……..………………………………………………*B. geminatus* (Li and Wang)

#### 3.2.3. Distribution

**Figure 1:**
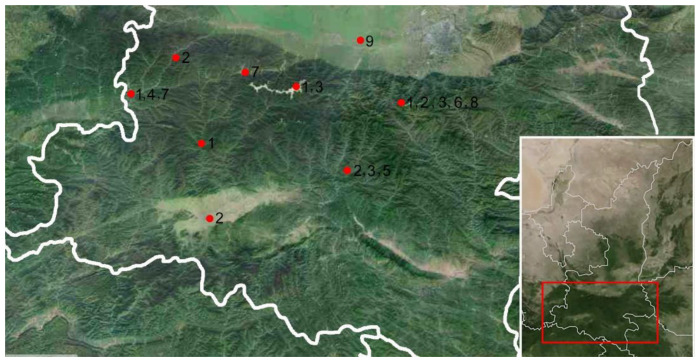
Geographical distribution of *Batracomorphus* species in Qinling Mountain of Shaanxi. As shown in the figure: 1. *B. allionii* (Turton); 2. *B. pandarus*; 3. *B. subfuscus* (Li and Wang); 4. *B. juno* Knight rec. nov.; 5. *B. fletcheri* sp. nov.; 6. *B. lateprocessus* Li and Wang; 7. *B. clavatus* Cai and Shen; 8. *B. lunatus* Cai and He; 9. *B. geminatus* (Li and Wang).

All nine species are found in two zoogeographical regions as in Figure 1. Among them, five species (*B. juno*, *B. clavatus*, *B. lateprocessus*, *B. lunatus* and *B. geminatus*) are distributed in the northern slope of Qinling Mountain, with *B. fletcheri* Hu and Dai sp. nov. only occurring in the south slope. Of the remaining species, *B. allionii*, *B. pandarus* and *B. subfuscus*, are from the southern to the northern slope. This study extends the known ranges of several species of the genus.

In the following section, detailed information on the collected specimens is reported with comments, and the descriptions of nine species are provided.

### 3.3. Species Description

#### 3.3.1. *Batracomorphus* Allionii (Turton, 1802)

**Figure 2:**
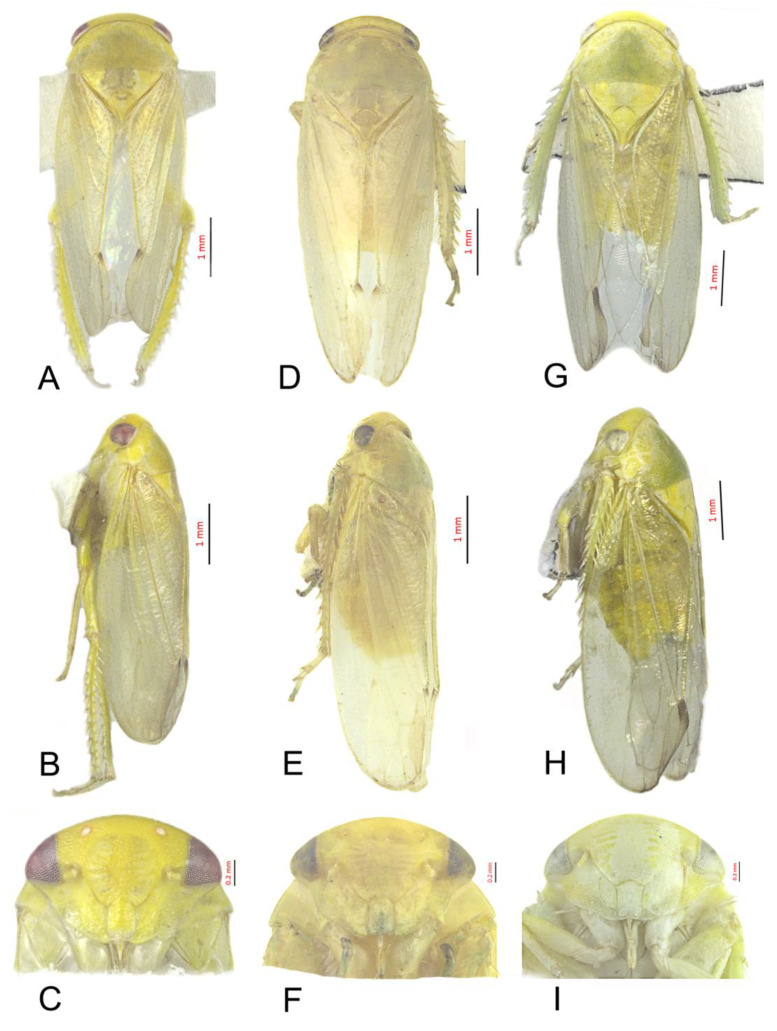
*Batracomophuspandarus* Knight, 1983: (**A**) habitus, ventral view, (**B**) habitus, lateral view, (**C**) face; *Batracomorphus allionii* (Turbon, 1802): (**D**) habitus, ventral view, (**E**) habitus, lateral view, (**F**) face; *Batracomorphus subfuscus* (Li and Wang, 1993): (**G**) habitus, ventral view, (**H**) habitus, lateral view, (**I**). face.

Figure 2D–F and Figure 5A–H.

*Cicada prasina* Fabricius, 1794: 38 [27] (Prim. hom.: *Cicada prasine* Pallas, 1773).

*Cicada allionii* Turton, 1802: 594 [28].

*Tettigonia prasine*, Latreille, 1804: 322 [29].

*Jassus prasinus*, Germar, 1821: 81 [30].

*Batracomorphus prasinus* (Fabricius): Lindberg, 1923: 69 [31]; Ossiannilsson, 1981: 355, figs. 1150–1158 [32].

Batracomorphus angustior Jacobi, 1943: 30 [33].

*Batrachomorphus* [sic] *prasinus* (Fabricius): Ribaut, 1952: 444, figs. 1178–1183 [34].

*Batrachomorphus* [sic] *fabricii* Metcalf, 1955: 266 [35], nov. pro. *Cicada prasina* Fabricius, 1794 [nec *Cicada prasina* Pallas, 1773].

*Batracomorphus fabricii* Metcalf, 1955: 266 [35], n. nov. pro *Cicada prasina* Fabricius, 1794, nec Pallas, 1773; Vilbaste, 1965: 31 [36].

*Batracomorphus allionii*, Metcalf, 1966: 121 [37]; Vilbaste, 1968: 62 [38]; Nast, 1987: 580 [39]; Anufriev and Emeljnaov, 1988: 85, Figure 57(7) [40]; Cai and Shen, 1998: 243 [41]; Cai et al., 2001: 187 [42]; Nickel 2003: 101 [43]; Cai and Shen, 2010: 17 [21]; Malenovsky and Lauterer, 2012: 228 [44]; Dai et al., 2013:149 [45]; Zhang et al., 2017:189 [46].

*Iassus dentatus* Kuoh, 1986: 83 [20]; Cai and Shen, 2001: 187 [42].

*Iassus trunctus* Li and Wang, 1993: 17 [18].

*Batracomorphus dentatus*, Li and Wang, 2003: 130 [19].

*Batracomorphus trunctus*, Li and Wang, 2003: 130 [19]; Cai and Shen, 2010: 17 [21].

Description. Male 5.2–6.8 mm long, 1.0–1.3 mm wide across eyes, 2.0–2.5 mm wide across hind margin of pronotum; Female 7.0–8.0 mm long.

Pale stramineous to pale yellowish green, with fuscous spot at base of appendix.

Pygophore narrowed to round caudal margin, inner process slender and straight through most of length, abruptly curved ventrally near apex, apex shallowly concave. Sub-genital plate semi-membranous, elongate, with a multiseriate row of long hair-like setae on ventral margin near mid-length and a short uniseriate row of long hair-like setae on dorsolateral margin near mid-length. Style narrowed near middle, slightly expanded distally with mesal margin serrate, apex hook-like. Aedeagus slender, dorsoatrium relatively long, approximately 2/3 length of aedeagus shaft, shaft with conspicuous lamellate lateral expansion subapically and lamellate extensions on anterior margin; gonopore extending approximately one-third length of shaft; apical incision short, approximately one-third length of gonopore.

Material examined. 4 ♂, Shaanxi, Fengxian County, 8.vii.1980, 798 m, Sun Hong; 1 ♂, Shaanxi, Liuba County, Miaotaizi, 19.vii.1995, Zhang Wenzhu and Ren Liyun, 1 ♂, Shaanxi, Taibai Mountain, vii.2002, 1170 m, Dai Wu (Figure 1).

Distribution. China (Heilongjiang, Jilin, Liaoning, Inner Mongolia, Shaanxi, Zhejiang, Henan, Hubei, Anhui, Shandong, Yunnan, Guizhou, Sichuan), Austria, Azerbaijan, Belgium, Czech, Slovakia, Denmark, Finland, France, Germany, Italy, Kazakhstan, Latvia, Lithuania, Mongolia, Netherlands, Norway, Poland, Romania, Russia, Spain, United Kingdom, Ukraine.

Remarks. *B. allionii* is mainly distributed in Europe. This species was originally described by Fabricius [27] from Italy as *Cicada prasina* and transferred to *Batracomorphus* by Lindberg [32]. Metcalf [36] found this name was a homonym and proposed the replacement name *fabricii*. Later, Metcalf [38] synonymized *B. fabricii* with *B*. *allionii* (Turton, 1802). Recently, two Chinese species, *B. dentatus* (Kuoh) and *B. trunctus* (Li and Wang) was considered to be a synonym with *B. allionii* (Turton) by Cai and Shen [21]. Based on the description and the illustrations of male genitilia provided by Ribaut [35] and Ossiannilsson [33], Chinese specimens we examined should be *B. allionii*.

Hosts. *Cytisus scoparius* and *Genista tinctoria*, and perhaps additional species of woody Fabaceae, and bamboo.

#### 3.3.2. *Batracomorphus pandarus* Knight, 1983

Figure 2A–C and Figure 6A–G

*Batracomorphus pandarus* Knight, 1983: 109, figs. 369–373 [8]; Wang et al., 2010: 168, figs. 1–3 [11].

Description. Male 4.0–5.5 mm long, 0.9–1.1 mm wide across eyes, 1.8–2.2 mm wide across hind margin of pronotum. Female 5.0–6.0 mm long, 0.9–1.1 mm wide across eyes, 1.9–2.2 mm wide across hind margin of pronotum.

Colour pale stramineous. Forewing with appendix at base piceous.

Pygofer side tapering gradually to blunt rounded margin, with many macrosetae near posterior margin, inner process moderately slender, arched near base then straight to ventromedially bent and tapered apex. Sub-genital plates semi-membranous, elongate, with a multiseriate row of long hair-like setae on ventral margin near mid-length and a short uniseriate row of long hair-like setae on dorsolateral margin near mid-length. Style simple, sinuate, width nearly uniform throughout length; apex hooked anterolaterally. Aedeagus with shaft slender, directed dorsally with apical one-fourth curved antero dorsally, tapering to apex in both lateral and posterior aspect, anterolateral margins produced flange-like apically and turned anteriorly. Gonopore extending approximately two-fifths length of shaft, apical incision approximately 3/4 length of gonopore.

Material examined. 1 ♂, Shaanxi, Baoji, Jialingjiang Park, 6.viii.2017, Zhu Qing and Xu YiFei; 2 ♂, Shaanxi, Baoji, Jialingjiang Park, 9.viii.2017, Zhu Qing, Xu YiFei; 1 ♂, Shaanxi, Baoji, Jialingjiang Park, 10.viii.2017, Zhu Qing, Xu YiFei (at light); 3 ♂, 1 ♀, Shaanxi, Ningshan, Huoditang Forest Station, 17–19. vii.2018, Sun Qihan; 1 ♂, Shaanxi, Ningshan, Huoditang Forest Station, 18.vii.2018, Xu Zhixin; 9 ♂, Shaanxi, Ningshan, Huoditang Forest Station, 20.viii.2018, Xu Yifei; 5 ♂, Shaanxi, Zhuque Forest Park, 22~25.vii.2007, Dai Wu; 1 ♂, Shaanxi, Zhuque Forest Park, 22~25.vii.2007, Yao Xia; 1 ♂ 2 ♀, Shaanxi, Hanzhong, 14.viii.2012, Gao Min (Figure 1).

Distribution. China (Shaanxi, Xinjiang), Borneo.

Remarks. This species was described by Knight in 1983 [8], based on three male specimens from Borneo. It is very similar to *B. romulus* Knight [8] but can be differentiated from the latter by the more robust aedeagal shaft and lack of the lateral flange-like expansion at the base of the shaft.

#### 3.3.3. *Batracomorphus* *subfuscus* (Li and Wang, 1993)

Figure 2G–I and Figure 7A–G

*Iassussubfuscus* Li and Wang, 1993: 18, Figure 4A–E [18].

*Batracomorphusnigromarginattus* Cai and Shen, 1999: 37 [47]; Cai, He and Gu, 2001: 187 [42]; Fang and Wu, 2001:34 [48]; Zhang et al., 2017:190 [46]. syn. nov.

*Batracomorphussubfuscus*, Li and Wang, 2003: 131 [19].

Description. Male 5.8–7.9 mm long, 1.2–1.3 mm wide across eyes, 2.1–2.2 mm wide across hind margin of pronotum.

Colour pale yellowish. Forewing with appendix piceous.

Pygofer side longer than height, tapering to acute apex, with many macrosetae near posterior margin, inner process moderately slender, extended straight posterodorsad to mid-length then bent posteroventrad, ventral margin serrate near apex, apex acuminate. Style thin, abruptly narrowed and sinuate in distal 1/4, apex hooked ventromesad. Connective stem gradually expanded posteriorly. Sub-genital plate thin, with many long fine setae along both margins. Aedeagus simple, shaft elongate, slender in lateral aspect, curving anterodorsally, laterally expanded in distal 1/3 subapically in posterior aspect, apical incision very short.

Material examined. 4 ♂, Shaanxi, Ningshan, Huoditang Forest Station, 17–19.vii.2018, Sun Qihan; 6 ♂, Shaanxi, Ningshan, Huoditang Forest Station, 18–19.vii.2018, Xu ZhiXin; 4 ♂, Shaanxi, Ningshan, Huoditang Forestry Station, 18.vii.2018, Xu YiFei; 3 ♂, Shaanxi, Taibai Mountain, Haopingsi, 15.vii.2002, 1170 m, Dai Wu (Figure 1) (NWAFU).

Distribution. China (Shaanxi, Henan, Guizhou, Zhejiang).

Remarks. This species was described based on one male and two female specimens deposited in the collection of Guizhou University. The illustrations given by Li and Wang (1993) [18] were misleading. Dr. J.C. Xing checked the type specimen and confirmed that the male genitalia illustrated here agree with the male genitalia of the holotype. *B.* *nigromarginattus* was described from Henan by Cai and Shen (1999) [48]. Based on the descriptions and illustrations provided by Li and Wang (1993) [18] and Cai and Shen (1999) [48], we propose the synonymy of these two taxa. This species is similar to *B. allionii* (Turton) but can be distinguished by the forewings with a dark brown appendix, the aedeagal shaft curved dorsally near the apex, and the appendage of pygofer taper to apex.

#### 3.3.4. *Batracomorphus juno* Knight, 1983 rec. nov.

**Figure 3:**
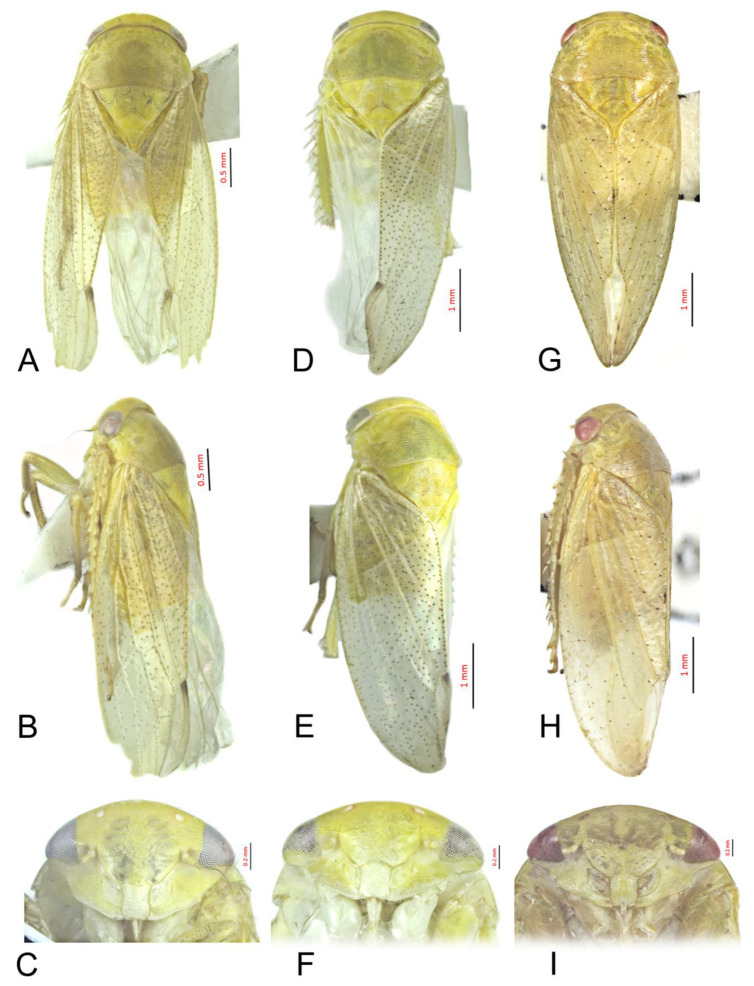
*Batracomorphusjuno* Knight, 1983 rec. nov.: (**A**) habitus, ventral view, (**B**) habitus, lateral view, (**C**) face; *Batracomorphus* *fletcheri* sp. nov.: (**D**) habitus, ventral view, (**E**) habitus, lateral view, (**F**) face; *Batracomorphus lateprocessus* Li and Wang, 2003: (**G**) habitus, ventral view, (**H**) habitus, lateral view, (**I**). face.

Figure 3A–C and Figure 8A–G

*Batracomorphus juno* Knight, 1983: 125, figs. 493–503 [8].

Description. Male 3.9–4.7 mm long, 0.9–1.0 mm wide across eyes, 1.6–2.0 mm wide across hind margin of pronotum.

Colour yellowish green. Forewings with distinctive fine setae and punctation black; appendix at base fuscous.

Pygofer side round apically, with many macrosetae near posterior margin, inner process slender, sinuate posteriorly, apex acute, curved posterodorsally, ventral margin acutely ridged subapically, sometimes with spur-like process, variable in relative length of process distad of spur. Sub-genital plate semi-membranous, elongate, with a multiseriate row of long hair-like setae on ventral margin near mid-length. Styles with apical process increasing gradually in width posteriorly, abruptly narrowed subapically to short acute dorsally hooked apex, ventral margin of subapical constriction acutely ridged. Aedeagus simple; shaft directed dorsally, tapered to apex over distal half; gonopore extending approximately two-thirds length of shaft; apical incision slightly shorter than gonopore, extending to near mid-length of shaft.

Material examined. 1 ♂, Shaanxi, FengXian, 7.viii.1980, 798 m, Sun Hong; 1 ♂, Shaanxi, FengXian, 15.vii.1995, Zhang Wenzhu and Ren Liyun (Figure 1). Other material. 1 ♂, Guizhou, 12.vii.2018, Zhu Qing (NWAFU).

Distribution. China (Shaanxi, Guizhou), Philippines, Borneo, and West Malaysia.

Notes: This species was described based on a series of specimens from Philippines, Borneo, and West Malaysia. It is similar to *B. spadix* Shen et Cai [21] but differs from the latter in having the style slightly curved in ventral aspect with apex hooked directed dorsally, and the pygophore processes slender and sinuate with the apex acute and directed dorsally, and exterior rim serrated.

#### 3.3.5. *Batracomorphus fletcheri* Hu and Dai sp. nov.

Figure 3D–F and Figure 9A–G

Description. Male 5.0 mm long, 1.0 mm wide across eyes, 1.9 mm wide across hind margin of pronotum.

Colour yellowish green. Forewings with distinctive fine setae and punctation black; appendix at base fuscous.

Pygophore protruding apically, with many macrosetae near caudal margin, inner process slender and evenly arched through most of length, broadened and recurved postero-dorsally near apex, apex foot-like with short, slender ventral heel and curved, tapered toe. Style thin near base slightly expanded beyond mid-length, unevenly tapered to dorsolaterally hooked apex. Sub-genital plate transparent and thin, long, with many long bristles on each margin. Aedeagus with shaft slender, elongate, directed dorsad and curved slightly anterad near apex, apex slightly narrowed in lateral view; parallel sided through most of length in posterior view; gonopore and apical incision relatively long, extending over one half of shaft.

Material examined. Holotype: ♂, Shaanxi, Ningshan, Huoditang Forest Station, 18.vii.2018, Sun Qihan (NWAFU) (Figure 1); Paratype: 1 ♂, Zhejiang, Tianmushan, 11.viii.1998 (at light) (NWAFU).

Distribution. China (Shaanxi, Zhejiang).

Etymology. This new species epithet honors Dr. Murray Fletcher, in recognition of his contributions to leafhopper studies.

Notes: This species is similar to *B. lineatus* Cai et Shen [21] but can be identified by the distinctive pygophore process with a foot-like apex, and the deeply divided apex of the aedeagus.

#### 3.3.6. *Batracomorphus* Lateprocessus Li and Wang, 2003

Figure 3G–I and Figure 10A–F

*Batracomorphuslateprocessus* Li and Wang, 2003: 134 [19].

Description. Male 6.3–6.8 mm long, 1.2 mm wide across eyes, 2.2 mm wide across hind margin of pronotum.

Color stramineous. Forewings with distinctive fine setae and punctation black.

Pygophore round apically, with many macrosetae near posterior margin, inner process moderately slender and nearly straight, slightly expanded in apical half, hardly reaching pygofer apex, tapering gradually to slender acute apex in lateral aspect. Style expanded over distal half and then tapering and gradually curved to upturned finger-like apex. Sub-genital plate semi-membranous, elongate, with stem and lateral lobe basally; a group of long hair-like setae on dorsolateral margin of lobe; a short uniseriate row of long hair-like setae on dorsolateral margin near mid-length; a multiseriate row of long hair-like setae on ventral margin near mid-length, extending also over medial surface of plate. Aedeagus simple, strongly enlarged and expanded ventrally at base; shaft relatively short, slender, arising from dorsal part of atrium, evenly recurved anteriorly, tapering to apex; evenly tapered in posterior aspect; gonopore extending approximately 1/2 length of shaft, apical incision very short.

Material examined. 1 ♂, Shaanxi, Zhuque Forestry Park, 22~25.vii.2007, Dai Wu (Figure 1). Other material: 1 ♂, Hunan, Hupingshan, 20.vii.2006, 1040 m, Lv Lin; 2 ♂, Zhejiang, Tianmushan, 27~26.viii.2000, Dai Wu and Wei Cong (NWAFU).

Distribution. China (Shaanxi, Zhejiang, Hunan)

Remarks. This species was described based on a male and a female specimen from Yunnan. Unfortunately, the illustrations given by Li and Wang (2003) [19] were misleading. Dr. Xing (personal communication) confirmed that the male genitalia illustrated here agree with the male genitalia of the holotype. This species resembles *B. thetis* Knight [8], but can be distinguished by the slender aedeagal shaft with the apex slightly curved anteriorly, and an apical process of style expanding over the distal half; the apical incision is very short.

#### 3.3.7. *Batracomorphus* *clavatus* Cai and Shen, 2010

**Figure 4:**
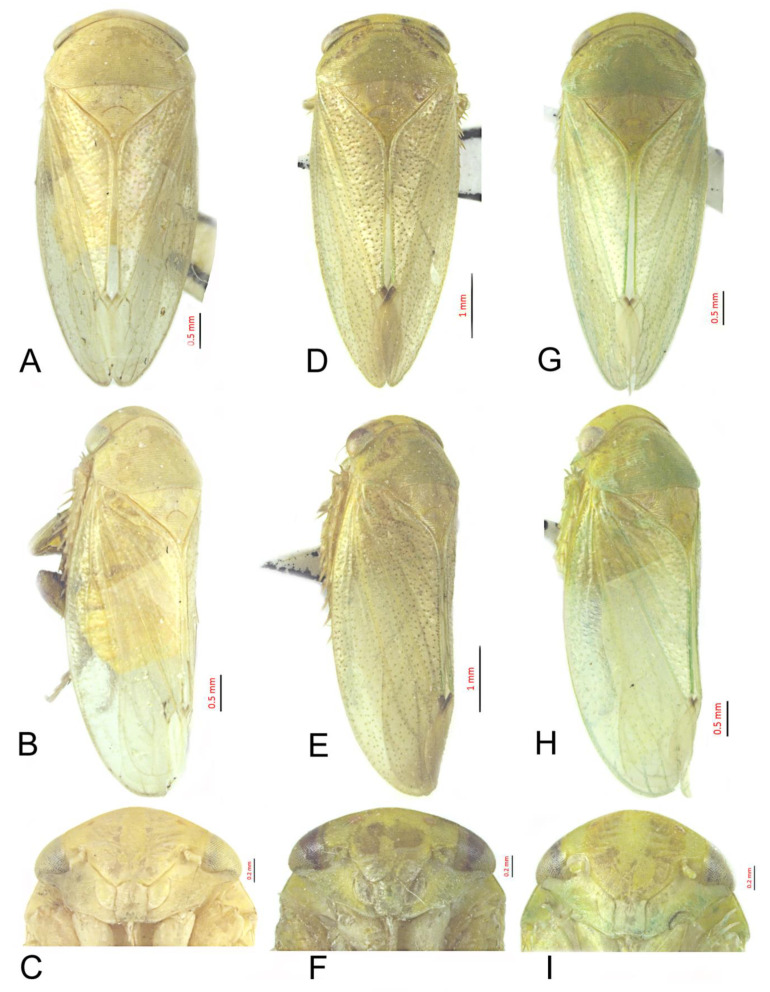
*Batracomophus****c****lavatus* Cai and Shen, 2010: (**A**) habitus, ventral view, (**B**) habitus, lateral view, (**C**) face; *Batracomorphus lunatus* Cai and He, 2001: (**D**) habitus, ventral view, (**E**) habitus, lateral view, (**F**) face; *Batracomorphus geminatus* (Li and Wang, 1993): (**G**) habitus, ventral view, (**H**) habitus, lateral view, (**I**) face.

**Figure 5:**
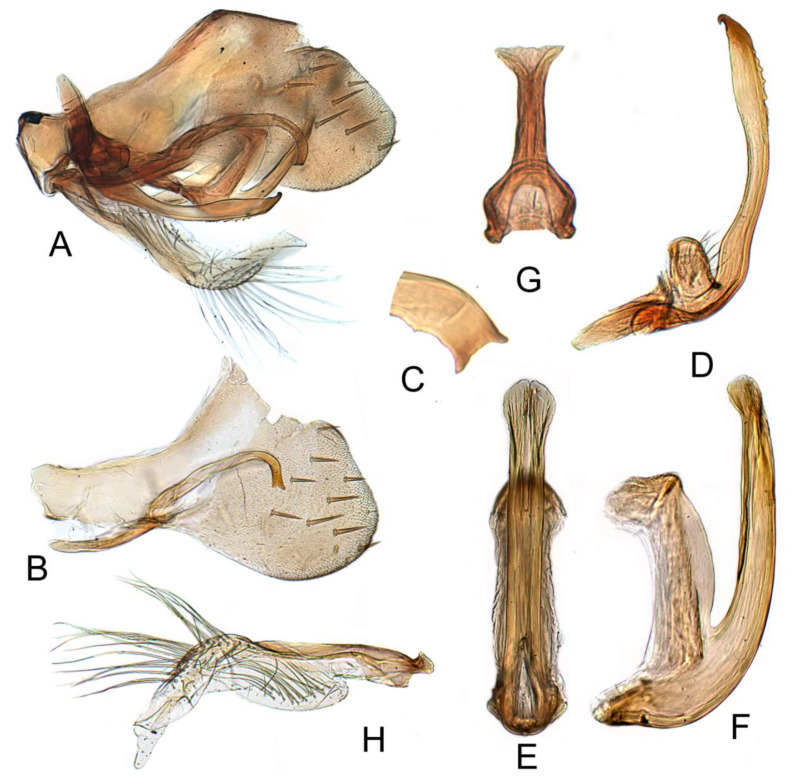
*Batracomorphus allionii* (Turton, 1802): (**A**) genital capsule in lateral view; (**B**) pygofer side; (**C**) pygophore processes apex; (**D**) styles; (**E**) aedeagus, ventral view; (**F**) aedeagus, lateral view; (**G**) connective; (**H**) sub-genital plate.

**Figure 6:**
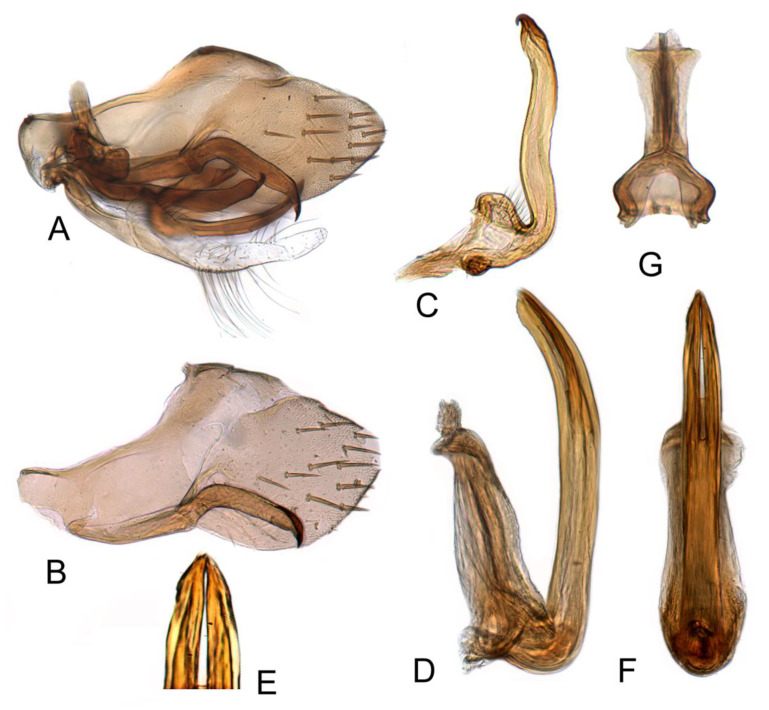
*Batracomophus pandarus* Knight, 1983: (**A**) genital capsule in lateral view; (**B**) pygofer side and pygophore processes; (**C**) styles; (**D**) aedeagus, lateral view; (**E**) aedeagus apex, ventral view; (**F**) aedeagus, ventral view; (**G**) connective.

**Figure 7:**
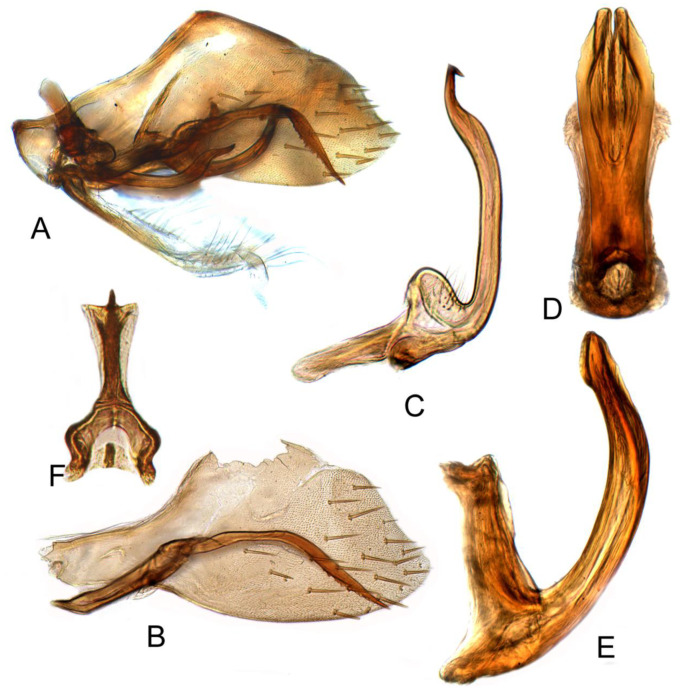
*Batracomorphus subfuscus* (Li and Wang, 1993): (**A**) genital capsule in lateral view; (**B**) pygofer side and pygophore processes; (**C**) styles; (**D**) aedeagus, ventral view; (**E**) aedeagus, lateral view; (**F**) connective.

**Figure 8:**
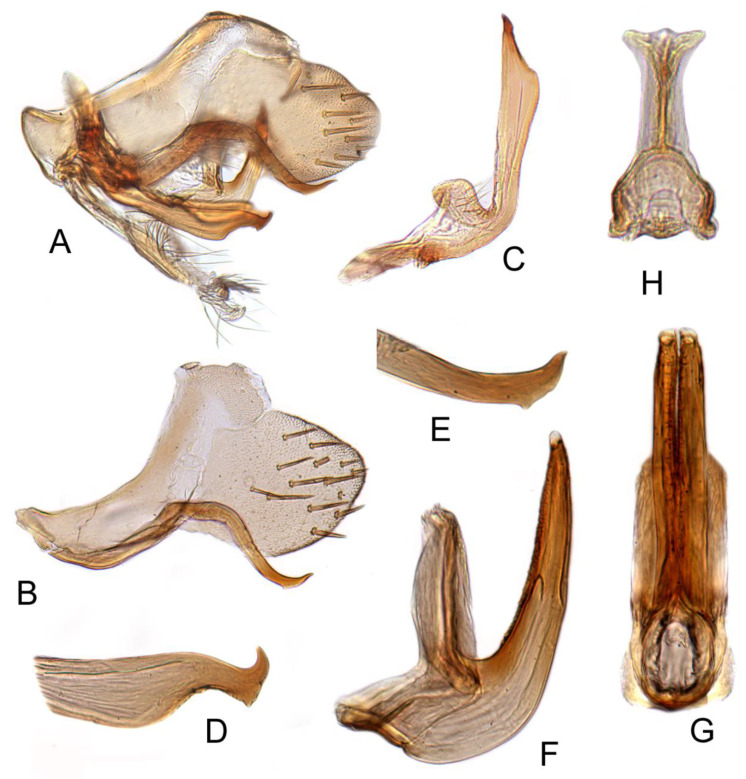
*Batracomorphus juno* Knight, 1983: (**A**) genital capsule in lateral view; (**B**) pygofer side and pygophore processes; (**C**) styles; (**D**) styles apex; (**E**) pygophore processes apex; (**F**) aedeagus, lateral view; (**G**) aedeagus, ventral view; (**H**) connective.

**Figure 9:**
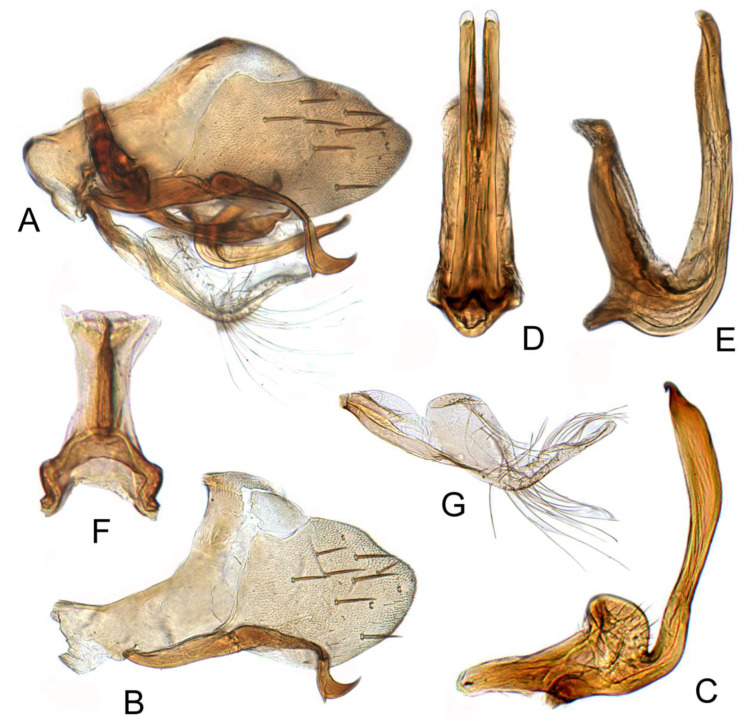
*Batracomorphus fletcheri* sp. nov.: (**A**) genital capsule in lateral view; (**B**) pygofer side and pygophore processes; (**C**) paranere; (**D**) aedeagus, ventral view; (**E**) aedeagus, lateral view; (**F**) connective; (**G**) sub-genital plate.

**Figure 10:**
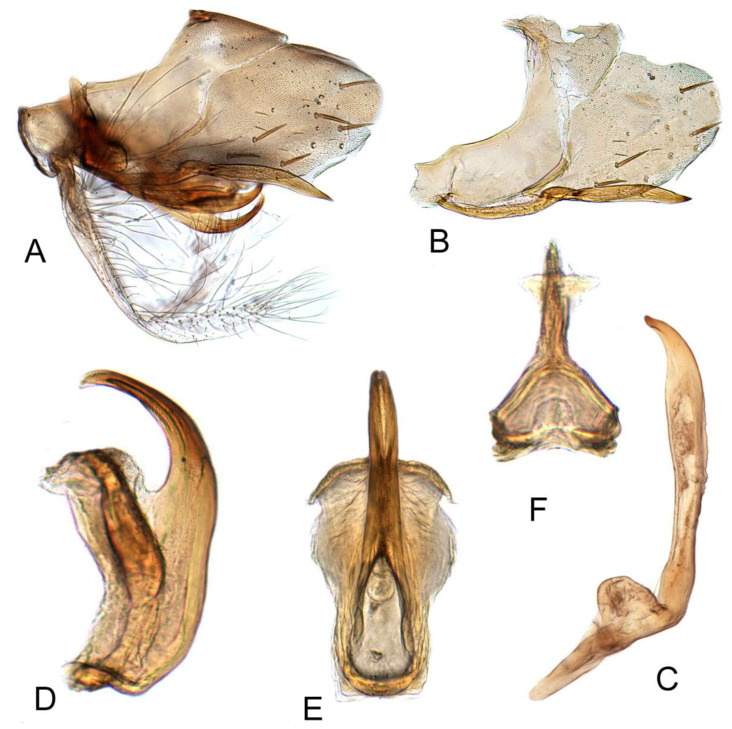
*Batracomorphus lateprocessus* Li and Wang, 2003: (**A**) genital capsule in lateral view; (**B**) pygofer side and pygophore processes; (**C**) styles; (**D**) aedeagus, lateral view; (**E**) aedeagus, ventral view; (**F**) connective.

**Figure 11:**
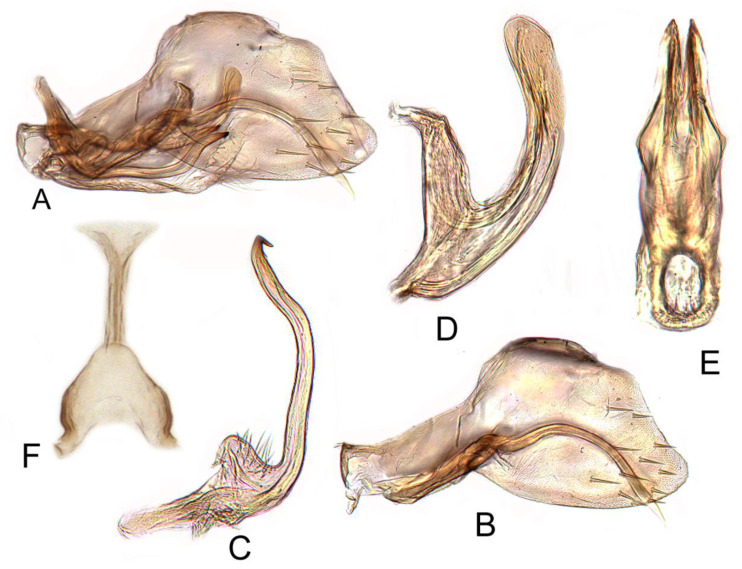
*Batracomorphus clavatus* Cai and Shen, 2010: (**A**) genital capsule in lateral view; (**B**) pygofer side and pygophore processes; (**C**) styles; (**D**) aedeagus, ventral view; (**E**) aedeagus, lateral view; (**F**) connective.

Figure 4A–C and Figure 11A–E

*Batracomorphusclavatus* Cai and Shen, 2010: 14, Figure 2A–G [21].

Description. Male 5.2–5.6 mm long, 0.9–1.1 mm wide across eyes, 1.9–2.0 mm wide across hind margin of pronotum. Female 5.5–6.3 mm long, 0.9–1.1 mm wide across eyes, 1.8–2.3 mm wide across hind margin of pronotum.

Colour stramineous. Forewing with appendix at base fuscous.

Pygophore protruding apically, distributed with sparsely short and thick bristles subapically, inner process slender, evenly arcuate, curving medially, tapering gradually to acute apex, ventral margin serrate near apex. Sub-genital plate semi-membranous, elongate, with uniseriate row of long hair-like setae on dorsolateral margin near mid-length and a multiseriate row of long hair-like setae on ventral margin near mid-length. Style long, slender, tapering gradually to acute dorsomedially hooked apex. Aedeagus with shaft short, evenly wide through most of length in lateral aspect, broadly rounded apically, directed posteriorly, and curved dorsally; in posterior aspect with lateral mar gins near mid-length expanded forming lamellate triangular flange; gonopore subapical at ventral margin.

Material examined. 3 ♂ 3 ♀, Shaanxi, Taibai, 14.viii.1981; 2 ♂ 2 ♀, Shaanxi, Taibai, 12.vii.1984; 2 ♂ 1 ♀, Shaanxi, Fengxian, 14.viii.1995, Zhang Wenzhu and Ren Liyun (Figure 1) (NWAFU).

Distribution. China (Shaanxi, Henan).

Remarks. This species was described by Shen and Cai [21] based on a male and a female specimen from Henan. It is similar to *B. iulus* Knight [8] but can be distinguished by the aedeagus with lamellate triangular flange at lateral margins near mid-length.

#### 3.3.8. *Batracomorphus lunatus* Cai and He, 2001

**Figure 12:**
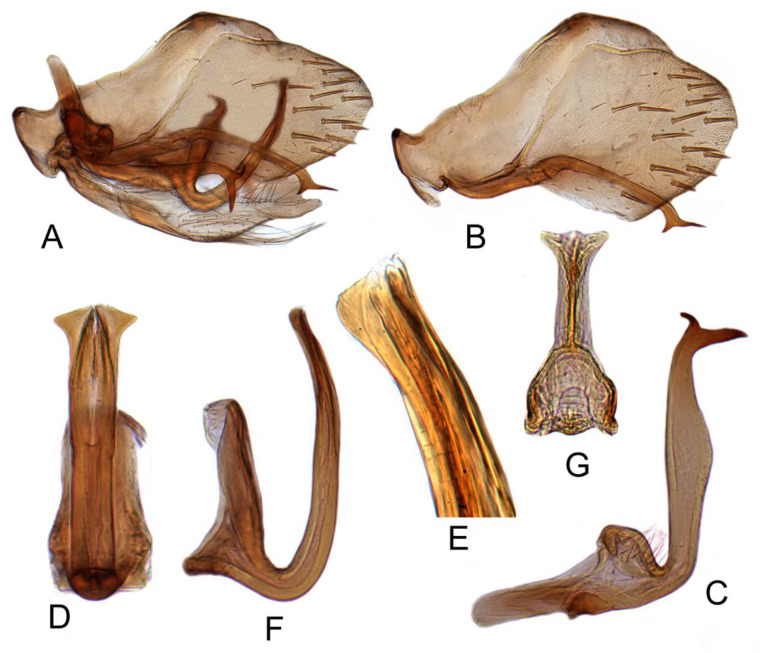
*Batracomorphus lunatus* Cai and He, 2001: (**A**) genital capsule in lateral view; (**B**) pygofer side and pygophore processes; (**C**) styles; (**D**) aedeagus, ventral view; (**E**) aedeagus apex, lateral view; (**F**) aedeagus apex, lateral view; (**G**) connective.

Figure 4D–F and Figure 12A–G

*Batracomorphus lunatus* Cai and He [in Cai, He and Gu, 2001], 2001: 191, figs. 20–25. [42]; Li et al., 2012:193 [49]; Fang and Wu, 2001:34 [48]; Zhang et al., 2017, 189 [46].

Description. Male 5.5–6.0 mm long, 1.0 mm wide across eyes, 1.9 mm wide across hind margin of pronotum.

Colour stramineous. Forewings with distinctive fine setae and punctation black; appendix at base fuscous.

Pygophore narrowed gradually to rounded caudal margin, with many macrosetae near posterior margin, inner process slender, elongate, arched dorsad at base, then straight through most of length to slightly decurved apex; with small subapical spur on ventral margin, portion of the process distad of spur usually longer than spur. Style slightly expanded at mid-length, tapering gradually to short acute ventromesally hooked apex, with small expansion subapically on dorsal margin. Sub-genital plate semi-membranous, elongate, with stem and lateral lobe basally; a group of long hair-like setae on dorsolateral margin of lobe; a short uniseriate row of long hair-like setae on dorsolateral margin near mid-length; a multiseriate row of long hair-like setae on ventral margin near mid-length. Aedeagus with shaft in lateral aspect slender, elongate, directed dorsally with apical third curving anterodorsally, in ventral view slightly wider; lateral margin over distal one-seventh expanded as lamellate triangular flange; gonopore extending to just basad of mid-length of shaft; apical incision short.

Material examined. 1 ♂, Shaanxi, Zhuque Forestry Park, 22~25.vii.2007, Dai Wu (Figure 1). Other material: 24 ♂, Zhejiang, Tianmushan, 25.vii.2011, 1500 m, Wang Yang (at light) (NWAFU).

Distribution: China (Guizhou, Shaanxi, Zhejiang).

Remarks: *B. lunatus* was described by Cai and He [42] from Zhejiang, China. This species is similar to *B. peteos* Knight [8] but differs from the latter in having the style and pygofer processes with subapical expansions, and the aedeagus with lamellate angular flanges apically. This species also resembles *B. romulus* Knight [8] but is distinguished by the aedeagus with a lamellate angular flange apically, and the style and pygofer processes with subapical expansions.

#### 3.3.9. *Batracomorphus geminatus* (Li and Wang, 1993)

**Figure 13:**
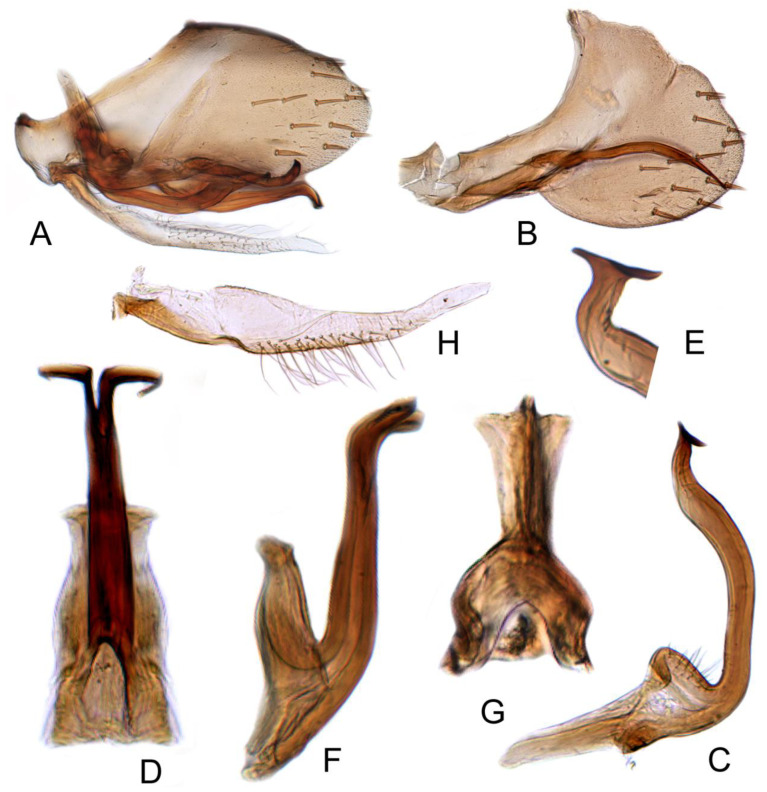
*Batracomorphus geminatus* (Li and Wang, 1993): (**A**) genital capsule in lateral view; (**B**) pygofer side and pygophore processes; (**C**) styles; (**D**) aedeagus, ventral view; (**E**) styles apex aedeagus, ventral view; (**F**) aedeagus, lateral view; (**G**) connective; (**H**) sub-genital plate.

Figure 4D–F and Figure 13A–H

*Iassus geminatus* Li and Wang, 1993: 18 [18].

*Batracomorphus geminatus*, Li and Wang, 2003: 131 [19].

Description. Male 5.6–6.0 mm long, 1.2 mm wide across eyes, 2.0 mm wide across hind margin of pronotum. Female 6.2 mm long.

Color yellowish green. Forewing with appendix at base fuscous.

Pygophore narrowing gradually to rounded caudal margin, with many macrosetae near posterior margin, inner process elongate, slender, arched throughout length, apex acute. Sub-genital plate transparent and thin, long, with fewer and short bristles born on each of side margins. Style with approximately uniform width to mid-length, then slightly widened to arched distal half, terminating in acute dorsally directed apex and with elongate ventrally directed projection subapically on ventral margin. Aedeagus with shaft slender, elongate, directed posteriorly and turning posteroventrally at sub-apex, terminating in a pair of divergent elongate processes directed laterad, with apices curved dorsally; gonopore subapical at ventral margin, apical incision very short.

Material examined. 1 ♂, Shaanxi, Wugong, 19.vi.1982 (at light) (Figure 1). Other material: 1 ♂, Hainan, Wuzhishan, 3.iv.2008, 600 m, Men Qiulei (NWAFU).

Distribution. China (Shaanxi, Guizhou, Hainan).

Remarks. This species was described based on a male and a female specimen from Yunnan. Unfortunately, the illustrations given by Li and Wang (1993) [18] were misleading. Dr. Xing (personal communication) confirmed that the male genitalia illustrated here agree with the male genitalia of the holotype. This species resembles *B. enyo* Knight [8] but differs from the latter by elongate pygophore processes and the aedeagus curved posteroventrally with long apical process directed laterally and then curved dorsally.

## 4. Conclusions

Nine species of the leafhopper genus *Batracomorphus* from Shaanxi are reviewed based on comparative morphological study, including one new species and one new synonym. This study extends the known ranges of several species of the genus. This study reveals considerable variation in several structures, especially the pygophore processes and aedeagus. Compared with the aedeagus, the pygophore processes are more variable which may provide a new focus for further research.

The Qinling Mountain belongs to a warm temperate zone and subtropical climate, which is suitable for species of the genus *Batracomorphus* mainly distributed in the tropical and subtropical environment. Species of *Batracomorphus* are mainly distributed on the northern slope from the Figure 1. Since a few samples were collected this time, the statistical results of leafhopper species may be affected. Follow-up research on leafhopper diversity from Qinling Mountain need to collect more specimens for confirmation.

## Data Availability

Data sharing not applicable.
